# Jean-Marc Reyrat (29/04/1967–28/10/2009)

**DOI:** 10.1111/j.1365-2958.2010.07049.x

**Published:** 2010-03

**Authors:** Nathalie Winter, Camille Locht, Rino Rappuoli

**Affiliations:** 1 INRAU1282, Centre de Tours, 37380 Nouzilly, France; 2 INSERM, Institut Pasteur de Lille1 rue du Professeur Calmette, 59000 Lille, France; 3 Novartis Vaccines and DiagnosticsSiena, Italy


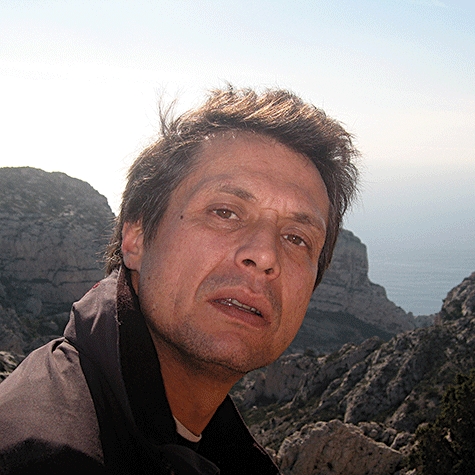


Microbiologists everywhere are saddened by the recent loss of Jean-Marc Reyrat, one of their most valued colleagues, who died after a short but brave battle against cancer. Qualities that come to mind when one remembers Jean-Marc are enthusiasm, strong motivation for scientific research, original thinking, critical assessment of his own work, integrity, fairness, loyalty and dedication to young scientists.

Jean-Marc fell in love with bugs in the early 1990s while studying Microbiology in Toulouse University, where he made his first steps in research in Pierre Boistard's lab. There, Jean-Marc dissected out regulatory mechanisms of nitrogen fixation by the symbiotic bacterium *Rhizobium meliloti* (Reyrat *et al.*, 1993; 1994;). His love for bacteria broadened thereafter to include many different types.

After completing his PhD in 1993, Jean-Marc went for a long trip in the Middle-East (back-packing was one of his passions) before joined Brigitte Gicquel's group at the Institut Pasteur in Paris to work on one of the major infectious agents affecting mankind, *Mycobacterium tuberculosis*. At that time, this slow growing, GC-rich bacterium was genetically almost totally intractable, and the scientific community doubted whether it would ever be possible to create null mutants. Here, one of Jean-Marc's characteristic traits came into play: never listen to what people say, just try yourself. Taking the urease gene of *Mycobacterium bovis* Bacille Camette-Guérin as a model, Jean-Marc was the first to demonstrate the feasibility of knocking out a gene in slow-growing mycobacteria (Reyrat *et al.*, 1995). Together with Vladimir Pelicic, then a PhD student in the same laboratory, Jean-Marc decided to test the possibility of using the *Bacillus subtilis sacB* gene encoding levansucrase as a counter-selectable marker. They reasoned that although mycobacteria are generally referred to as Gram-positive bacteria, their complex cell wall creates an outer membrane and, hence a periplasmic-like space that could render them sensitive to accumulation of levan produced from sucrose by levansucrase in the inter-membrane compartment. This prediction was validated, and the strategy was used to create null mutants of members of the *M. tuberculosis* complex. This powerful genetic tool was soon put to use by many mycobacterial geneticists to improve the selection of gene-replacement events in slow-growing mycobacteria. These major breakthroughs were reported in seminal papers (Pelicic *et al.*, 1996a,b;) and opened the way for the construction of allelic exchange mutants to identify *M. tuberculosis* virulence determinants.

Jean-Marc then decided to shift to another pathogenic bacterium, *Helicobacter pylori*, the major cause of stomach ulcers and strongly associated with gastric cancer. In 1996, he obtained a Marie Curie fellowship to join the renowned vaccine research centre of the Chiron Corporation – today Novartis – directed by Rino Rappuoli in Siena. He joined the team supervised by John Telford and studied the vacuolating cytotoxin, a major virulence factor encoded by the *vacA* gene in pathogenic *H. pylori*. In the 5 years that he spent in Siena, he made major contributions to the understanding of the function, cell specificity and the complex 3D structure of the VacA cytotoxin (Pagliaccia *et al.*, 1998; Reyrat *et al.*, 1999). Jean-Marc enjoyed the ‘dolce vita’ under the wonderful Tuscan sky.

After obtaining a permanent position in INSERM, the French National Institute of Health and Medical Research, Jean-Marc returned to the Institut Pasteur in 1998 to work again on mycobacteria. In 2003, he joined Xavier Nassif's INSERM unit at the Necker Faculty of Medicine, little over a stone's throw from the Institut Pasteur in Paris, as a young independent scientist in the highly competitive INSERM Avenir award scheme to develop his research project on *Mycobacterium smegmatis*, a non-pathogenic species that shares some genetic traits with pathogenic *M. tuberculosis*. The gamble was to use *M. smegmatis* as a model to dissect the molecular pathways for the synthesis of complex lipids that form a major component of the mycobacterial envelope (Reyrat and Kahn, 2001). Jean-Marc was very successful in this GAME (Genetic Analysis of the Mycobacterial Envelope) and published a series of important papers describing the pathways involved in the biosynthesis of some key molecules involved in communication with the host cell, such as glycopeptidolipids (Deshayes *et al.*, 2005; Sondèn *et al.*, 2005; Provvedi *et al.*, 2008). Recently, he turned to *Mycobacterium abscessus*, an emerging pathogen in cystic fibrosis patients that produces similar small key lipid molecules (Medjahed and Reyrat, 2009).

Jean-Marc was promoted Directeur de Recherche by INSERM in 2008 and was to be appointed to head his own INSERM team starting in 2010. Jean-Marc was a brilliant scientist, keen to understand the genetic basis of virulence in important human pathogens, as illustrated by his list of publications and the recent edition, with Mamadou Daffé, of a book on the *Mycobacterial Cell Envelope* (Daffé and Reyrat, 2008). However, restricting recognition of his dedication to science to the publication of papers in high-ranked journals would be unfair. Jean-Marc always found time to share with young people his enthusiasm for microbes and genetics. He created a course for young scientists to teach the use of bioinformatic tools. Probably the most prestigious achievement along this line was the ‘Young Scientist Symposia’ that Jean-Marc and his colleagues created in 2005. This 1 day congress was so well appreciated that five sessions were organized since 2005. The meeting this year, on September 29, was the last time Jean-Marc attended this gathering of young scientists that he initiated. One month later, Jean-Marc, aged 42 years, left us. We miss him deeply.
